# Archean crust and metallogenic zones in the Amazonian Craton sensed by satellite gravity data

**DOI:** 10.1038/s41598-019-39171-9

**Published:** 2019-02-22

**Authors:** J. G. Motta, C. R. de SOUZA FILHO, E. J. M. Carranza, C. Braitenberg

**Affiliations:** 1 Institute of Geosciences, University of Campinas, Rua Carlos Gomes, 250, 13083-855 Campinas, São Paulo Brazil; 2 University of KwaZulu-Natal, Westville Campus, Durban, 4001 South Africa; 3 Department of Mathematics and Geosciences, University of Trieste, Via E. Weiss 1, 34128 Trieste, Italy

## Abstract

The formation of ore deposits has been extensively studied from a shallow crust perspective. In contrast, the association of mineral systems with deep crustal structure of their host terranes remains relatively undisclosed, and there is evidence that processes throughout the lithosphere are coupled for their evolution. The current debate centers on the control of the regional deep crustal architecture in focusing and transferring fluids between geochemical reservoirs. Defining such architecture is not unequivocal, and involves combining indirect information in order to constrain its physical properties and evolution. Herein, based on evidence from satellite gravity, constrained by airborne potential field data (gravity and magnetics), we provide an example on how the lithosphere geometry controlled the location of copper and gold systems in the world-class Archean Carajás Mineral Province (Amazonian Craton, South America). Validation with information from passive seismic (wave speeds, crustal and lithospheric thickness) and geochronologic data (model, crystallization ages, and Neodymium isotope ratio determinations) portrays a significantly enlarged, poly-phase, Archean crust that exerted geometric control on the location of the mineral systems within and adjacent to the province during tectonic quiescence and switches. This new geologic scenario impacts the understanding of the Amazonian Craton. Synergy between multi-source data, as experimented here, can provide robust models efficiently and, conceivably, help to unveil similar terrains elsewhere.

## Introduction

Cratons are fragments of ancient continental crust containing complex records of the Earth’s evolution^1–4^, which often have been subjected to geological processes leading to the installment of mineral systems^5,6^. There is ample evidence from deposit- to regional- scale studies^7,8^ pointing out to a continuum of processes throughout the lithosphere to sustain these mineral systems^5,9^. However, it is contentious to ascertain the role of the whole crust architecture due to our limited capability of examining the deep crust^8^. In this context, recent advances on satellite gravity surveying offer a prosperous and yet untested tool^10,11^. Our focus here is on validating the interpretation of satellite gravity data using supporting information (airborne gravity and magnetics, passive seismics, model and crystallization ages) to model the crustal structure and constrain the development of an Archean mineral system within the Amazonian Craton in South America.

**Figure 1:**
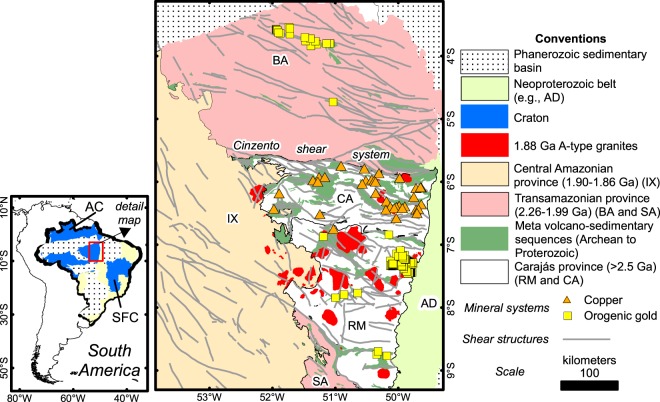
Simplified geological map of South America and details on the major geological framework of Brazil (left) and the south-eastern Amazonian Craton (detail, to the right). The detailed map shows the Archean Carajás Mineral province given by the Rio Maria (RM), Carajás (CA) domains, the Palaeoproterozoic Transamazonian province with the Bacajá (BA), and Santana do Araguaia (SA) domains, the Central Amazonian province with the Iriri-Xingu domain (IX), and the <850–757 Ma Araguaia domain (AD, a low-grade fold and thrust belt). AC: Amazonian Craton. SFC: São Francisco Craton.

Most of the information regarding the evolution of the Amazonian craton relies on reconnaissance geochronology studies, which led to its interpretation as a series of Palaeo- to Mesoproterozoic metamorphic belts surrounding restricted Archean crust^12–20^ (see Supplementary Information Appendix Fig. S1 for details). That applies to its south-eastern portion (Fig. 1), along with the Rio Maria, Carajás and Bacajá tectonic domains (Fig. 1)^14,16,17^. Their evolution goes back to the Mesoarchean docking^21,22^ of the Rio Maria (>3.0–2.8 Ga)^17,23–25^ to the Carajás domain (2.8–2.5 Ga)^17,19,20,22,26–28^, consolidating the Carajás Mineral Province (CMP)^29–31^. The CMP was later involved in a craton-wide deformation event during 2.26–1.86 Ga (Transamazonian Orogeny)^12,14,17,32^, which established the high metamorphic grade Bacajá^17,18^ and Santana do Araguaia^17^, and non-metamorphosed Iriri-Xingu^12^ domains around the CMP (Fig. 1), with localized metamorphic imprint on the province^28^. However, there are no independent bodies of evidence^14,18^ that distinguishes between Carajás and Bacajá domains other than the metamorphism age inventory.

Mineral deposits in the CMP have formed during tectonic switches and limited quiescent periods from Archean to Palaeoproterozoic^29,30^ (see Supplementary Information Appendix Fig. S1). They range from world-class iron oxide-copper-gold^33^ and poly-metallic copper systems^30,33–37^ (both referred to here as copper systems) (Fig. 1), orogenic gold deposits^31^, giant iron ore deposits^38^, and other commodities (e.g., Mn)^29^. The copper systems, which sum up to 2120 Mt ore with 0.8–1.4% Cu and 0.28–0.86 g/t Au^33,39^, representing the second most significant iron oxide-copper-gold district in the world^39^, have been described only in the Carajás domain^33^ to date. These deposits occur in sites of increased structural complexity^36,37,40^ along with regional-scale braided shear systems^31,33^. The regional hydrothermal alteration footprint of the copper system is independent on host rock type^33^ and derives from the interaction among various proportions of sedimentary, crustal- and mantle-derived fluids^33–35,41^. Crustal melting at ca. 1.88 Ga in the CMP and Iriri-Xingu terrains^42,43^ led to widespread A-type bimodal magmatism, and formed new copper systems and promoted remobilization on existent ones^33^. Orogenic gold systems, in contrast, have been described only outside the Carajás domain^31^, taking place in high-angle structures that cross-cut greenstone-like successions of varied ages^31^. Their formation is inferred to relate to processes involved in the consumption of oceanic crust during the Mesoarchean and Paleoproterozoic in the Rio Maria and Bacajá domains, respectively. Current models for the mineral systems in the CMP^30,35^ imply structural (re-) activations within a stable plate interior^30,33,44^ or proximity to zones of crust consumption^31^. However, at the moment, there are no clues to the lithosphere geometry in the CMP and the plausible extent of the envisaged continental plates. As proposals for the evolution of the CMP and surroundings considered unilateral evidence from the surface perspective only, we conjecture that a thorough examination of the lithospheric structure of the CMP could clarify its tectonic settings and harness proper links on the location of the mineral deposits therein.

Here we present a prediction model derived from qualitative and quantitative (forward and inverse modeling) interpretation of potential field data (see Methods) to unveil the lithospheric structure of the CMP and surroundings. We test the conjecture that the lithosphere architecture can be interpreted from satellite gravity data. To assess the reliability of the satellite data, we interpret them in conjunction with airborne- gravity and magnetic data, leading to a predictor model for the crust geometry. By focusing on long-wavelength gravity signatures, we can look for unrecognized Archean lithosphere within the Paleoproterozoic belts. Passive seismology data are employed to explore independent physical aspects of the inferred model down to the mantle lithosphere. We also use geochronology constraints to pinpoint the temporal evolution of the region, leading to a valid crustal model.

The potential field data show that Rio Maria and Carajás domains have distinct gravity and magnetic responses, whereas, Carajás to Bacajá domains are indistinct. A V_s_-velocity map at 100 km depth indicates that Carajás and Bacajá domains are part of a single lithospheric entity rooted in the mantle, and the values in V_p_/V_s_ ratios suggest a similar bulk crustal composition for the two domains. Model and crystallization ages, and trends in crustal evolution, point towards a shared history among the Carajás to Bacajá domains at least up to 2.5 Ga, with contrasting trajectories after the Transamazonian Orogeny. Thus, by combining the knowledge of physical structure with temporal evolution controls, we were able to devise two lithospheric pieces - the Rio Maria and the Carajás crusts. The latter constitutes an enlarged and unnoticed Archean plate interior that provided the necessary continental interior setting for the copper systems and the crust boundaries for orogenic gold deposits to form in the CMP. The satellite data provided unprecedented insights into the lithospheric structure in an approach that can be exported to constrain other regions abroad.

## Results

### Potential field data modeling

**Figure 2:**
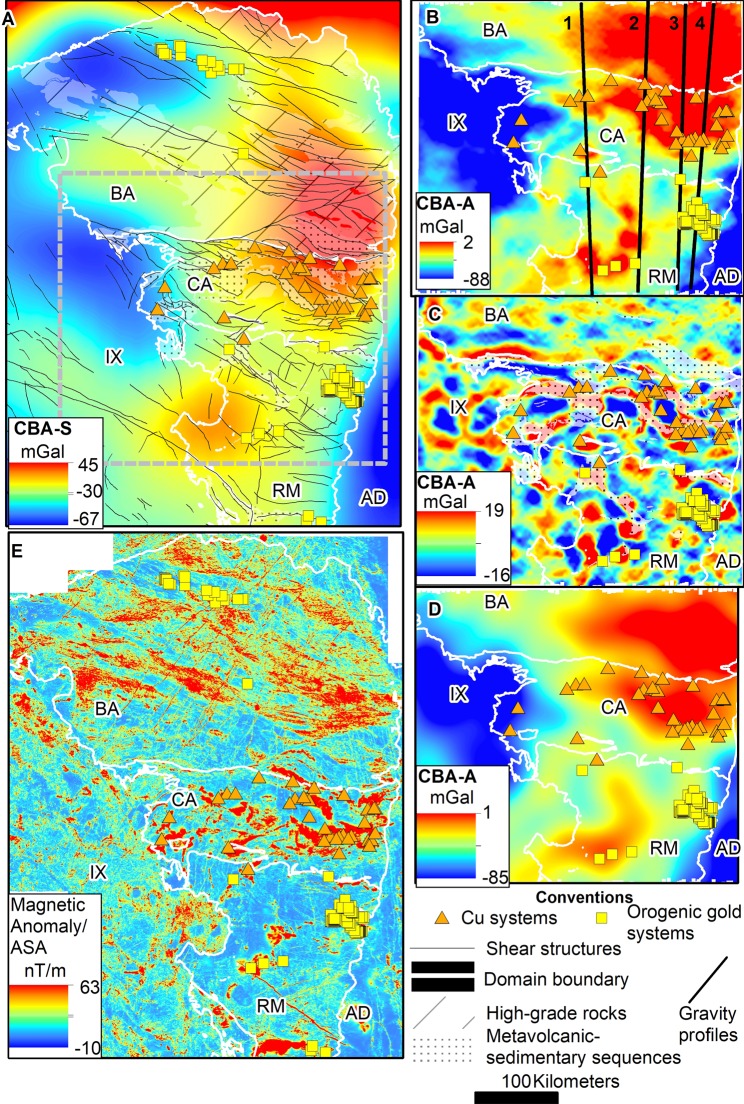
Products from airborne and satellite-borne potential field data. (**A**) Complete Bouguer anomaly map from the satellite gravity field model GOCO05s. The grey dashed line outlines the area surveyed by airborne gravity (in **B**,**C**,**D**). (**B**) Complete Bouguer anomaly map from airborne gravity data, filtered for causative bodies shallower than 10 km (**C**) and more profound than 10 km (**D**). (**E**) The amplitude of the analytic signal map extracted from airborne magnetic data. Profiles from 1 to 4 were forward modeled to constrain subsurface geometry (see Supplemental Information Appendix Fig. S2). High-gradient areas running in the NE-SW direction relate to Jurassic basic dikes. Acronyms: keys for domain names as in Fig. 1. CBA– complete Bouguer anomaly. S- satellite-borne. A – air-borne.

The complete Bouguer anomaly map derived from satellite gravity data (see Methods) shows a regional feature given by anomaly values ranging from −40 to 12 mGal running from the Rio Maria to Bacajá domains (Fig. 2A). The highest values (>−10 mGal) within the observed feature occur in the NE corner of the Carajás domain and follow into the Bacajá domain to the North, forming an oval shape. The anomaly value subdues to circa -24 mGal forming a broad zone trending WNW-ESE along the boundary from the Rio Maria to the Carajás domains. An abrupt decrease in the gravity anomaly (down to −30 mGal) exists in the central-northern part of the Bacajá domain, following an NW-SE trend (Fig. 2A). Values of the gravity anomaly tend to decrease towards both the Iriri-Xingu and Araguaia domains.

**Figure 3:**
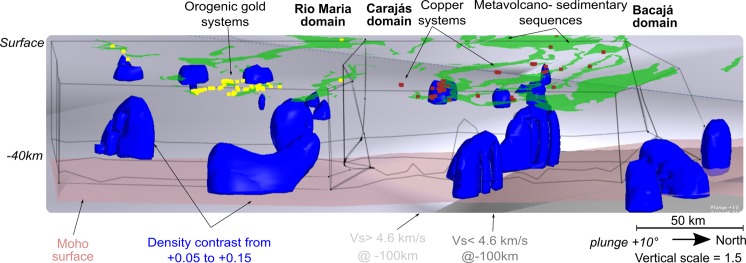
Three-dimensional model of the crustal infra-structure of the Rio Maria, Carajás and Bacajá domains from forward and inverse modeling of airborne gravity, with ancillary passive seismic and geological information. The infra-structure of the primary tectonic domains and Moho surface was yielded from forward models using airborne gravity data. Note the boundary in the volume for V_s_-speed^47^ close to the limit from Rio Maria to Carajás domains at 100 km depth.

The airborne gravity data (Fig. 2B) yield a similar picture as the satellite-borne data, although it details the interior of the tectonic domains. The broad regional high-anomaly zone in the airborne gravity data coincides with that in the satellite-borne data, both of which have well-defined E-W to WNW-ESE trends in their interior and reach two mGal within the Bacajá domain. The gravity features in the Carajás domain have a sigmoid shape trending E-W to NW-SE along the Cinzento shear system. These change gradually into linear structures to the north, following the trend in the Bacajá domain (Fig. 2B). The Rio Maria domain presents gravity anomalies up to −10 mGal, which distribute as belts with NW-SE, NE-SW, and E-W trends. The gravity anomaly subdues in an E-W trend close to the boundary between the Rio Maria and Carajás domains. The higher anomaly values within the domains are interpreted to relate to metavolcanic-metasedimentary sequences (e.g., Carajás Basin, Buritirama, Tapirapé and others in Carajás and Bacajá domains; Tucumã and Samambaia Groups in Rio Maria domain). These sequences interleave within the gneissic-granitic basement with subdued gravity response. It is evident from the results of spectral filtering and forward modeling of the airborne gravity data (see Methods) (Fig. 2C,D, Supplementary Information Appendix Fig. S2) that the metavolcanic-metasedimentary sequences responsible for anomaly gain are mostly restricted up to 10 km in depth and show two types of geometry. Firstly, they organize as roughly circular regions (Fig. 2C) amid the granite/gneissic bodies in the Rio Maria domain. Secondly, the sequences form an overall tabular geometry (Carajás Basin), with localized sub-vertical bodies along Neoarchean and Mesoarchean sequences at Carajás and Bacajá domains (Supplementary Information Appendix Fig. S2). For source bodies occurring deeper than 10 km (Fig. 2D) the field is similar to that noticed in the satellite gravity data (Fig. 2A). It is evident that the significant higher anomaly regions represent E-W to NW-SE-oriented trends oblique to the Cinzento shear system (Fig. 2D). A negligible anomaly contrast exists from the Carajás to the Bacajá domain. There is a sizeable subdued anomaly (up to −40 mGal) zone with E-W trend occurring from Rio Maria to Carajás that reaches the central part of the study area, close to the Carajás basin (Fig. 2D). These three tectonic domains were modeled as thick granitic-gneissic slices with high-angle boundaries among them (Supplementary Information Appendix Fig. S2, Fig. 3). The boundaries between the Rio Maria and the Carajás domain, and between the latter and the Bacajá domain, have no characteristic representation in the airborne gravity data. Given this, the three domains share similar gravity responses, despite their different crustal evolution and proposed subduction or accretion settings for their amalgamation^12,14,21,22^. The common features in gravity data of zones of past crust consumption through subduction and accretionary processes include: (i) the presence of paired belts of low- and high- gravity anomalies^45^ in a response of existing thickened and thinned crustal sections, respectively; and (ii) characteristic representation of crust over- thickening over the younger terrane^2,3,46^. Thus, gravity data modeling is not able to extract evidence of the past operation of a subduction zone setting between the Rio Maria and Carajás domains.

Inverse modeling of the airborne gravity data (see Methods) resolves the regions with high anomaly values (Fig. 2B,D) as a series of kilometer-scale, discontinuous causative bodies (Fig. 3). These arrange as near-vertical ovoid shapes with density contrasts varying from +0.10 to +0.19 g/cm³ (Fig. 3), which occur at depths greater than 15 km. They follow an NW-SE to E-W trend in Carajás and Bacajá domains, and, in contrast, a nearly N-S trend in Rio Maria domain (Fig. 3). The high-density bodies along the Cinzento shear system coincide with the thrust surface collocating the Bacajá over the Carajás domain (Fig. 3, Supplementary Information Appendix Fig. S2). Furthermore, the high-density bodies underlie the abundant metavolcanic-metasedimentary sequences in every domain and minor Archean-Paleoproterozoic basic-ultrabasic rocks in the Carajás domain (Fig. 3).

The 3D analytic signal amplitude (ASA) map derived from airborne magnetic data shows a subtle magnetic texture within the Rio Maria domain with low amplitude anomalies (<5 nT/m). Features form sinuous NW-SE, N-S, and E-W trends, the last being frequent in the east, along with the boundary to the Carajás domain (Fig. 2E). Conversely, the Carajás domain presents a texture of medium to higher gradients (>0.5 to 10 nT/m) oriented in NW-SE to E-W and NE-SW trends, with discontinuous forms (Fig. 2E). A prominent sigmoidal feature in central Carajás relates to abundant banded iron formations in the Carajás Basin (Fig. 2E). Northeast-southwest trending lines prevail in western Carajás. A change in the texture is observed from Carajás to Bacajá along the Cinzento shear system, where sinuous, broad features in Carajás turn into thinner, parallel, linear, high gradients in the Bacajá domain (Fig. 2E). This evidence suggests that any potential contact between the Carajás and Bacajá domains is gradual. The trends detected in the Rio Maria, Carajás, and Bacajá domains become less prominent in the Iriri-Xingu domain, being superposed by E-W to NE-SW low gradient features (Fig. 2E).

The observed patterns in the potential field data mark a division of the structural framework of the CMP from the Rio Maria to the Carajás domain, which, then, extends into the Bacajá domain. To constrain the predicted model against the inherent subjectivity and ambiguity of potential field analysis, we examine existing seismic constraints over the area for validation.

### Seismic constraints for the predicted framework

To constrain our predicted model for the crustal structure independently, we analyze the V_p_ and V_s_ velocity structure^47,48^, and the Lithosphere-Asthenosphere Boundary (LAB)^49^ depth in the region. The V_s_ -velocity from seismic tomography studies^47^ shows a velocity boundary within the mantle at 100 km depth (Fig. 3), which lies close to the limit between the Rio Maria to Carajás domains. The Rio Maria domain presents bulk V_s_ higher than 4.6 km/s, whereas Carajás and Bacajá domains show velocities lower than that. Accordingly, a common V_p_/V_s_ ratio (~1.7)^48^ is observed in the crust from Carajás to Bacajá (Supplementary Information Appendix Fig. S3), suggesting a similar bulk felsic crust for them. It is observed that the lithosphere is thicker in the Rio Maria domain (275, 0 km), being progressively thinner below the Carajás (250, 0–275, 0 km) and northward into the Bacajá domain (<225, 0 km) (Supplementary Information Appendix Fig. S3). This suggests that the Rio Maria resides over a deeper lithosphere, in accordance with its old and stable crustal evolution history. This contrasts with the lithospheric thinning observed throughout the Carajás and Bacajá domains, in accordance with their protracted evolution and modified lithospheric settings. Thus, the Carajás and Bacajá domains share properties on a broad scale from the crust to deep down into the mantle lithosphere, with Rio Maria presenting different overall properties from them (See Supplementary Information Appendix Fig. S3 for details). These observations align with evidence from potential field data.

### Crustal evolution constraints

We examine the geochronology inventory (depleted-mantle model - T_DM_, and crystallization ages, Neodymium isotope ratio determinations- εNd; see Methods) to evaluate the representativeness of the predicted physical model against the existent age inventory and current understanding of the CMP formation.

**Figure 4:**
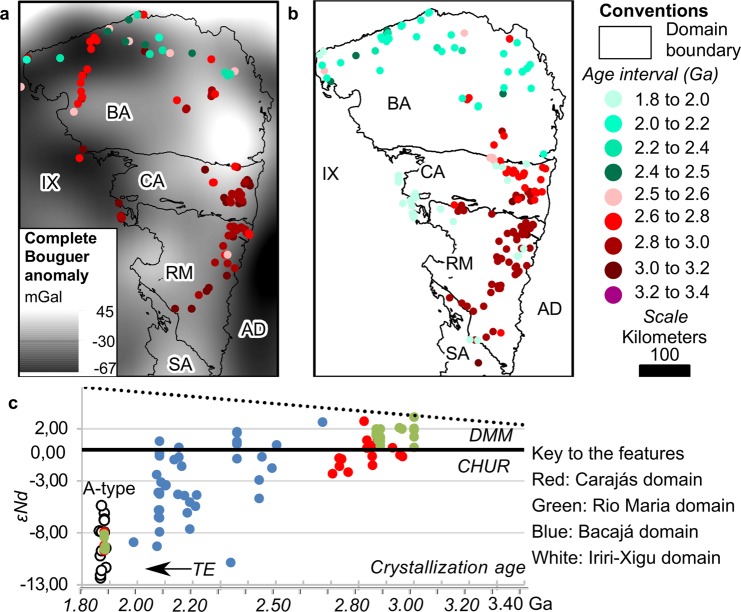
Geochronology inventory for the CMP and surroundings. (**a**) Sm-Nd T_DM_ model age determinations draped over the complete Bouguer anomaly derived from satellite data. (**b**) Protolith crystallization age determinations. (**c**) Crustal evolution diagram according to the depleted mantle model (DMM) and the chondritic uniform reservoir (CHUR). Keys for domain names are as in Fig. 1.

From the comparison of the observed regional gravity anomalies against the distribution of T_DM_ model ages (Fig. 4a), a set of observations emerges. It is noticeable that the observed higher values in the gravity anomalies coincide with a consistent set of ages ranging from 3.2 to 2.6 Ga. Furthermore, the abrupt decline in the gravity anomaly observed in the central-northern part of the Bacajá domain coincides with the existence of a set of ages younger than 2.5 Ga. Such younger ages occur as an NW-SE trending group that truncates the older age cluster (Fig. 4a). The T_DM_ ages in Carajás and Rio Maria vary within the range described above. However, ages older than 2.8 Ga up to 3.4 Ga (Fig. 4a) are more frequent. Restricted samples in the Iriri-Xingu domain align with the patterns observed in both the CMP and Bacajá domain.

By analyzing the spatial distribution of the crystallization ages, it is clear that ages from 3.4 to 2.5 Ga dominate in Carajás and Rio Maria, also being present in Bacajá (Fig. 4b). Crystallization ages younger than 2.6 Ga prevail north of the Cinzento shear system, and ages younger than 2.5 Ga prevail from the central to the northern part of the Bacajá domain (Fig. 4b). These younger ages depict Rhyacian arc-granitoids and basic rocks^12,50^ alongside scattered Archean determinations in the high-grade basement. The Carajás domain concentrates Archean ages up to 3.2 Ga, whereas ages older than 2.8 up to 3.3 Ga predominate in the Rio Maria domain (Fig. 4b).

From the crustal evolution diagram (Fig. 4c), it is apparent that the history of the Rio Maria domain is predominantly older than 2.8 Ga, with the addition of juvenile material (Fig. 4c). The Carajás domain has a predominant share of pre-2.5 Ga juvenile to poorly contaminated crust (mildly negative εNd values) (Fig. 4c). The Bacajá domain follows the same trend observed in the Carajás domain before 2.0 Ga. A broader spread in εNd values after the Archean where mildly positive εNd values in the Bacajá domain (Fig. 4c) represent the localized addition of juvenile crust or a mixture of juvenile and evolved crust in the Early Palaeoproterozoic occurring from the central to the northern part of the domain. Rocks with strongly negative εNd values prevail in the Bacajá from ca. 2.0 Ga onwards, which associate to the crustal reworking throughout the Transamazonian event. The Iriri-Xingu domain presents a similar crustal evolution pattern to the Bacajá domain (Fig. 4c). Abundant, strongly negative εNd values (<−8 to −12) (Fig. 4c) are associated with 1.88 Ga A-type granites restricted to the CMP and Iriri-Xingu domain.

By the examination of the T_DM_ and crystallization ages at the CMP and surroundings, it is observed that the Carajás and Bacajá domains share evolution stages before the Transamazonian event. The distribution of the common T_DM_ and crystallization ages is concordant to the physical model as derived from potential field data and validated by the seismic constraints. Regarding the plausible common history from Carajás to Bacajá domains, it is observed from their petrological and geochemical inventories that: i) both hold a predominantly magmatic nature for their gneissic-granitic basement with Archean protoliths;^18,19,50,51^ and ii) supracrustal protoliths are restricted to northern Bacajá domain^17,18^. This set of evidence allows considering a close evolution for Carajás and south-central Bacajá domains involving a similar Archean crust before the Transamazonian Orogeny.

## Discussion

**Figure 5:**
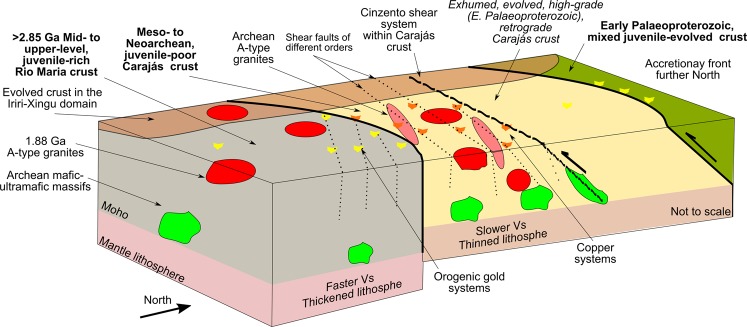
Interpreted crustal framework for the Carajás Mineral Province given by extended Archean lithosphere as interpreted from geophysical constraints on the crustal architecture and geochronology.

A set of clues about the crust structure and its temporal evolution opposes the separation of the Bacajá from the Carajás domain and endorses the individualization of the Rio Maria domain. The Supplementary Information Table 1 summarizes characteristics of the domains regarding physical structure and evolution. Based on the present evidence, we consider the regional architecture as two lithospheric elements that were juxtaposed before the Transamazonian Orogeny (Fig. 5) - the Rio Maria and Carajás crusts and their respective mantle roots. We consider two scenarios to reconcile the pre-Transamazonian association between Bacajá and Carajás domains. The first hypothesis takes the two domains as a continuous crust parcel as early as the docking of the Carajás to the Rio Maria crust in the Mesoarchean^21,22^. In this case, the Cinzento shear zone represents a pre-existing structure within the Carajás crust, being (re-) activated in the Transamazonian event^52^. Secondly, the Carajás and Bacajá domains share an undisclosed Archean history between the docking of the former to the Rio Maria crust and the Transamazonian orogeny. The formation of the Carajás crust would be given, then, by the juxtaposition of the Carajás and Bacajá proto-domains and its cratonization sometime before the Transamazonian Orogeny, along with the Cinzento shear system. That is of particular interest given the undisclosed settings of high-temperature crystallization circa 2.5 and 2.4 Ga^36^ within Mesoarchean gneisses along the Cinzento shear system. A plausible situation for a previous separation of the Carajás and proto-Bacajá domains within the Carajás crust would be that of continental rifting associated with the formation of the Carajás basin^44^. However, in the absence of a detailed description of the gneissic basement and analysis of protolith age inventory to the South of the Bacajá domain, we cannot ascribe a precise scenario.

In any of these scenarios, we argue that during the Transamazonian Orogeny the Cinzento shear system (Fig. 5) could have acted over (re-)activation of previous structures^52,53^ within a protracted Archean lithosphere. Thus, we reconcile these to an inward situation to an extended accretionary front, likely a back-arc setting during the Transamazonian Orogeny. In this way, exotic and Rhyacian juvenile rocks (e. g. <2.4 to 2.0 Ga felsic intrusive rocks^18,50^) concentrated along the edge of the Carajás crust to the north (Figs 4a,b, 5). Furthermore, we constrain the continental interior for the formation of the Carajás Basin as an intracontinental rift, as previously argued^44^. The high-density bodies on the infrastructure of the Rio Maria and Carajás crusts are interpreted as related to the Archean poly-phase basic/ultra-basic magmatism, likely forming the basal parts of the magmatic systems. Proposals claiming for an extended Archean framework within the Bacajá domain^16^ previously failed to provide evidence for this notion.

Our validated model reveals a previously unknown extension of the Archean substratum in the Amazonian Craton (Fig. 5). By extending the Archean substratum of the Carajás domain to the north, we offer insights into the ancient craton interior and infer that favorable areas for copper systems may extend further. Nevertheless, the mineral deposits, if preserved, would have been modified by the Transamazonian Orogeny. Given the enlarged crustal setting in the Carajás crust, we suggest that the copper systems formed in an intra-continental situation rather than proximal to collision settings during the Archean^33,35^. Also, the lack of clues for classic subduction from Rio Maria to Carajás precludes immediate comparison of the iron oxide-copper-gold copper systems and orogenic gold deposits to younger equivalents^54,55^. In any case, we argue that mineralizing fluids circulated with reduced connectivity between domains (Fig. 5), taking advantage of the mostly vertical discontinuities^30,31,33,34,52^. The large-scale permeability would account for the mixed signatures of the copper mineralizing fluids^30,33,34,41^. Regarding the overall constitution of the Amazonian craton, our model documents the entanglement of the deep crust in the Archean nucleus of the craton during the Transamazonian Orogeny, opposing the soft-collision tectonics view for its evolution^12^.

Our results show that modeling by integration of satellite gravity data to other geophysical data, and the validation of the model by supporting evidence from passive seismic and geochronological data can reveal aspects of the physical structure of large craton areas that are difficult to be tracked. The methodological approach followed here has significant practical applications to understand global geodynamic processes and inspection of mineralized crust elsewhere.

## Methods and Materials

### Potential field data compilation and processing

We interpret satellite-borne gravity data to evaluate the most profound density structure of the Amazonian Craton sector investigated here. Such data derive from the satellite-only Earth Gravity Observation Combination version 05 (GOCO05s)^56^ gravity model. The gravity data were taken as the magnitude of the gradient of the gravity potential calculated at 6,000 meters above sea-level, and truncated at degree/order 250 in the spherical harmonic model, as is the highest degree of GOCE-derived fields^56,57^. The truncation was necessary to avoid high-frequency noise^56^. Data were stored in a 0.2-degree mesh. A topography grid was extracted from the ETOPO1 Global Relief Model^58^ at same grid dimension, point density, and degree expansion to the gravity functional for use in topography-dependent processing. The processing was implemented in Python (v. 2.71) using the *Fatiando a Terra* library (version 0.4)^59^. The processing pipeline follows previously established procedures^11^. The first step is to calculate the normal gravity in its closed-form^60^. The normal gravity is then subtracted from the magnitude of the gradient of the gravity potential to obtain the gravity anomaly. The topography effect at each point was computed and removed from the gravity anomaly to correct the data for topographic effect. The topography effect was calculated by discretizing the topography with tesseroids (spherical prisms) and forward modeling their response. The densities in the continent and oceanic areas have different values (2670 kgm^−3^ and −1630 kgm^−3^, respectively), with calculation undertaken with the classical 167 km radius from observation points. The complete Bouguer anomaly data were then gridded with 0.05 degrees cell-size by minimum curvature interpolation^61^.

The airborne gravity was used to inspect the crustal structure at smaller scales than the long wavelength satellite gravity data. Airborne gravity data comprise a survey carried out from 2013 to 2014 by the Geological Survey of Brazil (CPRM) and designed with N-S-directed flight lines spaced by 3000 m and at 100 m ground clearance. Data are available as complete Bouguer anomaly values, reduced with the same densities and reduction radius as explained above. The Bouguer values were gridded into 600 m sided mesh using the bi-directional line gridding algorithm. To inspect the vertical distribution of the density sources, we used the power spectrum wavelength-decay technique^62^ over the complete Bouguer anomaly. The power spectrum reveals two source assemblies: i) from the surface to 10 km in depth, and b) from 10 to 35 km. By application of band-pass filtering, we isolated these two assembly levels into shallow (from the surface to 10 km, pass half-wavelengths smaller than 60 km) (Fig. 2C) and more profound (from 10 to 35 km, reject half-wavelengths smaller than 60 km) (Fig. 2D) into gravity anomaly maps for each level. The different spectra were isolated by high-pass and low-pass filters cut-off half-wavelength of 60 km. The forward modeling was carried out on the GM-SYS platform over the complete Bouguer anomaly from the airborne survey. The modeling software is part of the Geosoft Oasis Montaj suite and follows standard techniques^63,64^. Density values for modeling come from compilations of physical properties^65,66^, given lack of local samples. Profiles were modeled with constraints from lithology and structures in surface geology and for crustal thickness from seismic data^48,67^. The gravity product from the satellite model and the airborne survey were interpreted in their respective flight heights. The results of the application of the power spectrum technique over the airborne gravity data are not unique, given that the decay of the gravity signal of the source body is influenced by the size of the horizontal slabs that contain the bodies, their depth, and thickness^62^ and the inherent ambiguity of the gravity field^63–66^.

The airborne gravity data were subjected to inverse modeling targeting the density contrast distribution. The inversion was achieved using the Geosoft VOXI cloud-computing platform. The inversion algorithm minimizes the objective function (Iterative Reweighting Inversion Focusing) considering the misfit between observed and estimated anomalies with depth-weighing/smoothing functions. The inverted volume was discretized as rectangular prisms with dimensions of 10 × 10 × 5 km³ that define the voxels, with the smaller dimension in the vertical direction. The algorithm returns the physical property contrast to the reference density used in the calculation of the Bouguer anomaly. In the absence of external constraints, our inversion experiments were run by constraining the maximum depth on the solutions according to the power spectra checked in both cases and described above. We ran the inversion on the anomaly for both shallow (<10 km) and more profound (10–35 km) volumes separately and by limiting the inverted volume to these pre-defined boundary levels. The inverted prisms are limited to the crust, since the Moho from seismic investigations is deeper than 37 km, as constrained by seismology studies in the South American platform^48,67^. In any case, the inversion is one of the possible models that define the relative density inhomogeneity. These results were then used to make a refined forward model accounting for all available constraints and geologically driven assumptions on the existing density bodies.

Airborne magnetic data were used to find clues from the magnetic grain of the study area with complete resolution, in a way similar to the airborne gravity data. The magnetic data set comprises a compilation of 10 airborne surveys carried out by CPRM from 2004 to 2014. Individual surveys were carried out in N-S flights spaced by 500 m with 100 m ground clearance. The individual data were gridded to a standard cell size of 125 m using bi-directional line gridding algorithm. The grids were then stitched together by a suturing algorithm that corrects magnetic anomaly values to assure smooth transitions and de-noise the boundary regions. Further processing of the magnetic data comprised elimination of spurious short frequency by a low- pass filter with 50 m cut-off wavelength. Departing from the total magnetic anomaly map, we produced a 3-D analytic signal amplitude (ASA)^68–70^ map to highlight the edges of magnetized bodies^70^.

Filtering of the airborne potential field data and interpolation of processed data into maps were performed in the Geosoft Oasis Montaj Platform. Integration of the outcomes of potential field data by filtering, forward and inverse modeling and other auxiliary data were carried out in ESRI ArcGIS 10.5 for 2D surfaces, and Leapfrog Geo 4.0 for 3D surfaces and volumes.

### Seismic information

Information on the Moho discontinuity depth^48,67^, results from Vs^47^ - tomography from continental scale passive seismic investigations, and V_p_/V_s_^48^ ratio provide constraints on the velocity structure of the crust and mantle in the Amazonian Craton. Results for the Lithosphere-Asthenosphere Boundary (LAB) from the global lithospheric model LITHO1^49^ were examined to provide constraints on the structure of the underlying mantle. The lithosphere thickness has been used as a proxy for the evolution of the mantle roots of the overlying crust because the older, evolved lithosphere is usually thickened, being thicker over regions of older development rather than over regions that have been reworked or destabilized by different processes (e.g., orogenesis, intra-plate processes)^1,47–49^. Regarding this, we observed the characteristic depth of the LAB over the study area as a proxy from which to draw inferences on how the crustal domains, as observed from the surface through our geophysical examination and auxiliary data (e.g., geochemistry), connect to regions of different lithospheric thickness.

### Geochronological data

To constrain our interpretations over the physical structure of the terranes regarding feasible geological evolution, we evaluated their age relationships from a compiled geochronology data set. We examined the T_DM_ model and crystallization ages to unveil the relationships of parent material to a common ancestor reservoir, and crystallization of igneous and protoliths rocks, respectively. Evaluation of εNd determinations provide clues for the derivation of crustal material during crystallization and reworking episodes. The compiled data set comprises 414 age determinations from various minerals and methods (Sm-Nd, U-Pb, Pb-Pb, K-Ar, Ar-Ar isotopic systems). The target ages were model (n = 142) and crystallization (n = 272) ages. A total of 99 simultaneous determinations of Sm and Nd isotopic composition and crystallization ages with εNd calculations were compiled to evaluate crustal evolution. Data come from more than 50 publications, most of them cited in papers^14–18,21,22,26,28,30,31,33–35,42,50^ and references therein. We are aware that the geochronology data set is deficient in some aspects, such as the existence of geographical gaps (e.g., western Carajás, southern Bacajá), sample collection bias, and preservation of the geochronology record, or even incomplete inspection of the record.

### Geology data

To assess the geological meaning of the interpretations, we used published geology information from CPRM^17^. The datasets comprise lithological and structural maps and mineral deposit information. Mineral deposits locations come from publications about the copper systems^33,37^ and public exploration reports; the same applies for the orogenic gold systems^31^. A summary of geological, geophysical and geochronological features of the study area is given in Supplementary Information Table 1.

## Supplementary information

Supplementary information

## Data Availability

Data could be made available upon request to the corresponding author.
