# Supplementary material for: Archean crust and metallogenic zones in the Amazonian Craton sensed by satellite gravity data
Source: Sci Rep. 2019 Feb 22;9:2565. doi: 10.1038/s41598-019-39171-9 (PMC6385487; doi:10.1038/s41598-019-39171-9)
Supplement: Supplementary file 1 — Supplementary information [file 41598_2019_39171_MOESM1_ESM.docx]

**SUPPLEMENTARY INFORMATION**

**Title** - Archean crust and metallogenic zones in the Amazonian Craton sensed by satellite gravity data

**Authors** – J.G. Motta¹*, C. R. de Souza Filho¹, E. J. M. Carranza², C. Braitenberg³.

¹Institute of Geosciences, State University of Campinas, Campinas, São Paulo, Brazil. Rua Carlos Gomes, 250, Postal code 13083-855, Campinas, São Paulo, Brazil. ²University of KwaZulu-Natal, Westville Campus, Durban 4001, South Africa

³Department of Mathematics and Geosciences, University of Trieste, Via E. Weiss 1, 34128, Trieste, Italy.

The supplemental information appendix contains accessory information for the understanding of the regional context of the study area (Supplementary Information figures S1, S3) and extended results (Supplementary Information Figure S2, Supplementary Information Table 1).

**Supplementary Information Figure S1** – **Timeline chart for the evolution of the south-eastern Amazonian Craton.** Upper panel - mineral deposit formation stages of mineral deposits^1–5^; middle panel - geological events^6–11^. Periods of mineral deposits formation are marked with yellow squares for orogenic gold systems, orange triangles for copper systems, red squares for banded iron formations (BIF) deposition and upgrading. Lower panel - shear deformation episodes before or during the cratonization of CMP^4,12^, and reactivations in the Mesoproterozoic history ^4,12,13^. BA - Bacajá domain, BIF – banded iron formations, CA- Carajás domain, RM – Rio Maria domain, IX - Iriri-Xingu domain, MVSS - meta volcano-sedimentary sequences.

**
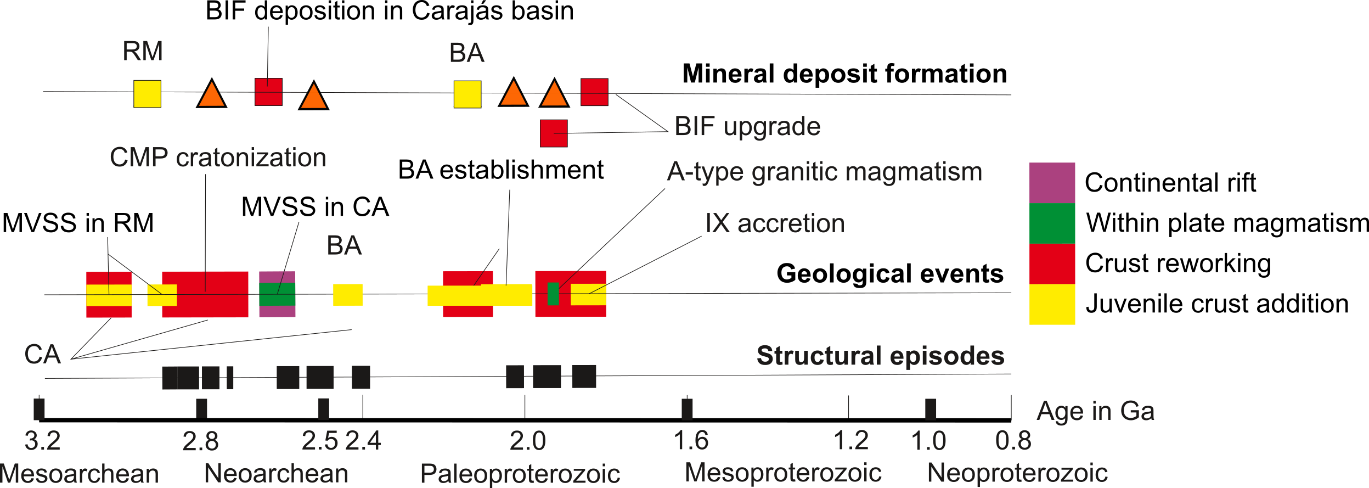
**

**Supplementary Information Figure S2** – **Forward modeling sections from the airborne gravity survey**. Profile positions are according to traces in Figure 2b. a) profile 1; b) profile 2; c) profile 3 and d) profile 4. The modeled outlines of Rio Maria, Carajás, Bacajá, the lower crust, and mantle were used on the integrated 3D model in Figure 4. Acronyms: XC: Xingú complex, BJO: Bom Jesus orthogneiss, AVT: Arco Verde tonalite, CT: Caracol tonalite, MT: Mogno trondhjemite, NIG: Nova Índia gneiss, SCM: São Carlos meta tonalite; TTG: trondhjemite-tonalite-granodiorite associations.


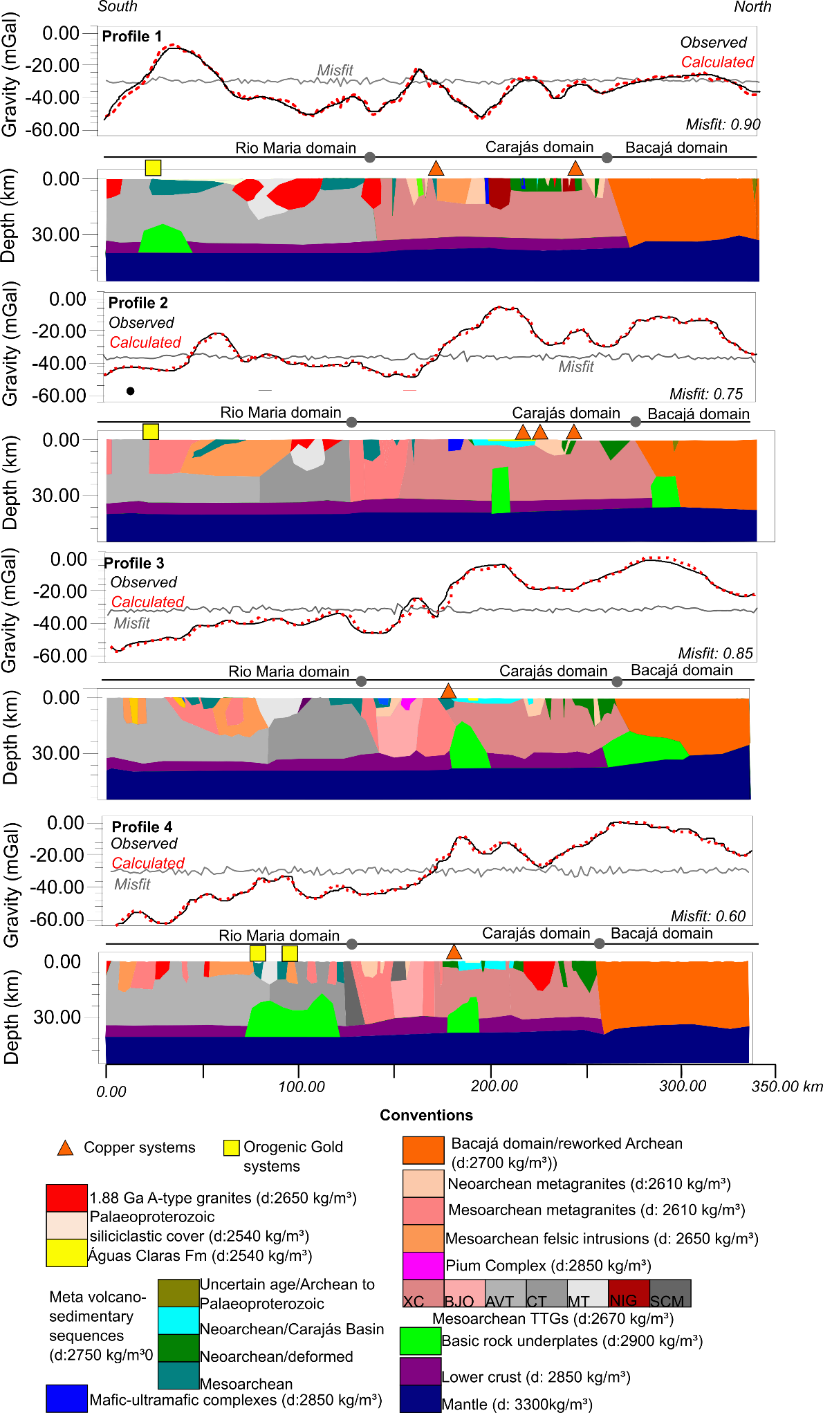


**Supplementary Information Figure S3** – **Seismology information over the Carajás Mineral Province and surroundings.** Contour map for the Lithosphere-Asthenosphere Boundary (LAB) depth from the LITHO1 global model^14^, and passive seismic stations with information on Moho depth and the V_p_/V_s_ ratio^15,16^.


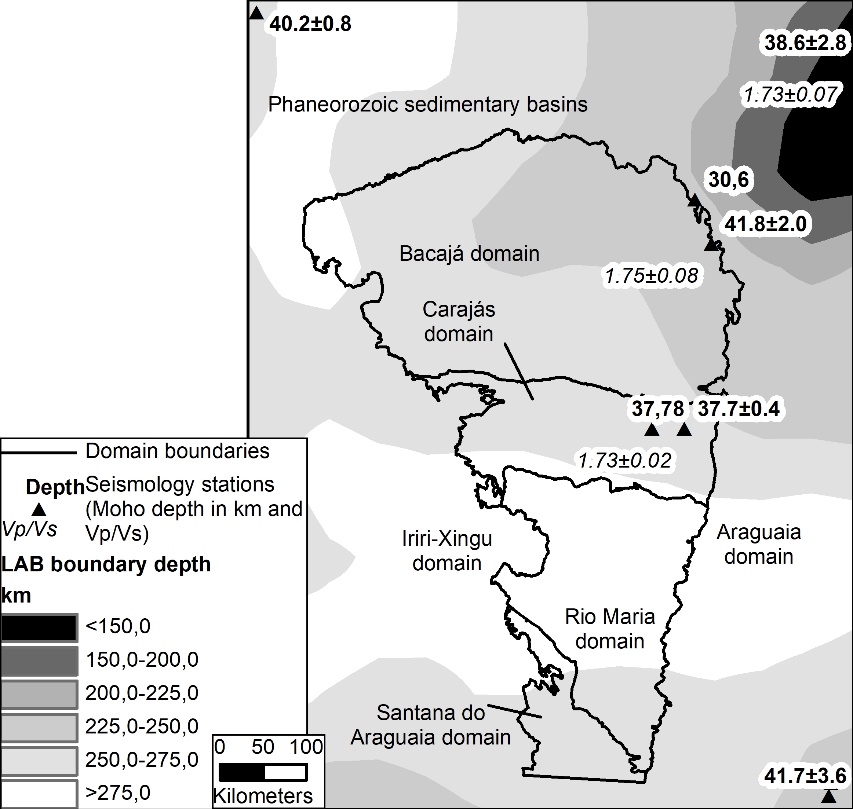


**Supplementary Information Table 1** - Summary table for the geology, geochronology and geochemistry/geochronology inventory of the Carajás Mineral province and its surrounding Palaeoproterozoic belts. Key: ASA – Analytical signal amplitude of the magnetic anomaly; LAB – Lithosphere-Asthenosphere boundary depth; T_DM_ – depleted mantle model ages from Sm-Nd isotope geochemistry; TTG – tonalite-trondhjemite-granodiorite association; S – satellite-borne gravity information; A) airborne-gravity information.

|  | **Rio Maria domain** | **Carajás domain** | **Bacajá domain** | **Iriri-Xingu domain** |
| --- | --- | --- | --- | --- |
| **Magnetic texture in ASA maps** | subtle low-gradient texture, mainly NW-SE high-gradient trends. Secondary NE-SW and E-W gradients. Overall low-gradient magnetic framework | Sinuous, discontinuous E-W, WNW-SSE and NE-SW(minor) high gradient zones | Sinuous, continuous NW-SE to E-W high-gradient zones, prominent to the North and NE. Discontinuous, sinuous, high-gradient fabrics from the center to the South which is transitional to the Carajás fabric. | NNW-SSE to NNE-SSW low-gradient fabric along a subtle magnetic framework. |
| **Density texture in Bouguer anomaly maps** | S) <10 mGal, rounded high-anomaly region to the South. A) discontinuous linear to rounded high-anomaly trends with NW-SE to NE-SW direction | S) regional high-anomaly region >-10mGal. A) subtle, discontinuous E-W to NW-SE high-anomaly sinuous shapes. Prominent high-anomaly region in the deep-sources anomaly maps up to 2 mGal. | S) regional high-anomaly region >-10mGal. A) subtle, discontinuous E-W high-anomaly sinuous shapes. The prominent high-anomaly region in the deep-sources anomaly maps up to 2 mGal. | S and A) overall low anomaly region (<-20 mGal). |
| **LAB thickness** | >275 km | 275-250 km | 275-225 km (rapidly thinning to the N-NE) | >250 km |
| **Wave-speed information** | Vs >4.6 km/s @ 100km | Vs <4.6 km/s @ 100km; Vp/Vs = 1.73±0.02 | Vs <4.6 km/s @ 100km; Vp/Vs = 1.75±0.08 | - |
| **Overall composition** | TTG + sanukitoid basement (orthoderived), volcano-sedimentary sequences (greenstone belts, komatiites are present) | TTG basement (orthoderived), volcano-sedimentary sequences (little- to no komatiites, chemical sediment-rich, ~2.7 Ga greenstone succession) | granite-gneissic basement (orthoderived from center to south; para-derived to the center to north), volcano sedimentary rocks are frequent (Archean to the South, Proterozoic from the center to North) | overall acid/intermediate volcanic, volcaniclastic and plutonic counterparts |
| **Structural framework** | discrete, linear, regional NW-SE shear zones, dome and keel granite-greenstone basement. | the patent, sinuous, E-W to WNW-SSE shear zones (poly-phase, Archean) | the patent, sinuous WNW-SSE to NW-SE shear zones, E-W reverse thrusts (footwall to the South) to the South, localized NE-SW shear faults. | NNW-SSE no E-W brittle faults |
| **T_DM_ age ranges** | 2.7 to 3.4 Ga | 2.7 to 3.2 Ga | 3.0 to 2.2 Ga | 3.0 to 2.2 Ga |
| **Crystallization age ranges** | 2.8 to 3.4 Ga | 2.5 to 3.0 Ga | 2.6 to 2.0 Ga | <2.0 Ga |
| **Metamorphism ages** | amphibolite facies (>2.8 Ga, basement and greenstone belt sequences) | granulite facies (>3.0 Ga) and amphibolite facies (<2.7 to 2.8 Ga) | amphibolite retrograde over granulite (Early Palaeoproterozoic) | not metamorphic |

**REFERENCES TO THE SUPPLEMENTARY MATERIAL**

1. Grainger, C. J., Groves, D. I., Tallarico, F. H. B. & Fletcher, I. R. Metallogenesis of the Carajás Mineral Province, Southern Amazon Craton, Brazil: Varying styles of Archean through Paleoproterozoic to Neoproterozoic base- and precious-metal mineralisation. *Ore Geol. Rev.* **33,** 451–489 (2008).

2. Villas, R. N. & Santos, M. D. Gold deposits of the Carajás mineral province: Deposit types and metallogenesis. *Miner. Depos.* **36,** 300–311 (2001).

3. Xavier, R. P. *et al.* The iron oxide copper-gold systems of the Carajás mineral province. *Econ. Geol.* **16,** 433–454 (2012).

4. Moreto, C. P. N. *et al.* Timing of multiple hydrothermal events in the iron oxide–copper–gold deposits of the Southern Copper Belt, Carajás Province, Brazil. *Miner. Depos.* **50,** 517–546 (2015).

5. Silva, R. C. F. e *et al.* Hydrothermal fluid processes and evolution of the giant serra norte jaspilite-hosted iron ore deposits, Carajás mineral Province, Brazil. *Econ. Geol.* **108,** 739–779 (2013).

6. Tassinari, C. C. G. & Macambira, M. J. B. Geochronological provinces of the Amazonian Craton. *Episodes* **22,** 174–182 (1999).

7. Vasquez, M. L. *et al.* *Geologia e Recursos Minerais do Estado do Pará: Sistema de Informações Geográficas - SIG: texto explicativo dos mapas Geológico e Tectônico e de Recursos Minerais do Estado do Pará*. (CPRM, Belém., 2008).

8. Macambira, M. J. B. *et al.* Crustal growth of the central-eastern Paleoproterozoic domain, SW Amazonian craton: Juvenile accretion vs. reworking. *J. South Am. Earth Sci.* **27,** 235–246 (2009).

9. Olszewski, W. J., Wirth, K. R., Gibbs, A. K. & Gaudette, H. E. The Age, Origin, and Tectonics of the Grao-Para Group and Associated Rocks, Serra-Dos-Carajas, Brazil - Archean Continental Volcanism and Rifting. *Precambrian Res.* **42,** 229–254 (1989).

10. De Souza, Z. S. *et al.* Nd, Pb and Sr isotopes in the Identidade Belt, an Archaean greenstone belt of the Rio Maria region (Carajás Province, Brazil): Implications for the Archaean geodynamic evolution of the Amazonian Craton. *Precambrian Res.* **109,** 293–315 (2001).

11. Dall’Agnol, R. *et al.* Petrogenesis of the Paleoproterozoic rapakivi A-type granites of the Archean Carajás metallogenic province, Brazil. *Lithos* **80,** 101–129 (2005).

12. Pinheiro, R. V. L. & Holdsworth, R. E. Reactivation of Archaean strike-slip fault systems, Amazon region, Brazil. *J. Geol. Soc. London.* **154,** 99–103 (1997).

13. deMelo, G. H. C. *et al.* Temporal evolution of the giant Salobo IOCG deposit, Carajás Province (Brazil): constraints from paragenesis of hydrothermal alteration and U-Pb geochronology. *Miner. Depos.* **52,** 709–732 (2017).

14. Pasyanos, M. E., Masters, T. G., Laske, G. & Ma, Z. LITHO1.0: An updated crust and lithospheric model of the Earth. *J. Geophys. Res. Solid Earth* **119,** n/a--n/a (2014).

15. Albuquerque, D. F. *et al.* Crustal structure of the Amazonian Craton and adjacent provinces in Brazil. *J. South Am. Earth Sci.* **79,** 431–442 (2017).

16. Assumpção, M. *et al.* Crustal thickness map of Brazil: Data compilation and main features. *J. South Am. Earth Sci.* **43,** 74–85 (2013).
